# Case of Fibro-Cartilaginous Tumor—Ovarian Cyst and Stricture of the Vagina—Death from Peritonitis

**Published:** 1864-12

**Authors:** Wm. Gould


ART. IV.—*Case of Fibro-Cartilaginous Tumor—Ovarian Cyst and Stricture of the Vagina—Death from Peritonitis. By* WM. GOULD, M. D.

As the following case may be of some interest to your readers, I propose to give a report of it from the time it first came under my notice. It embraces a period of some eight years. My notes, for obvious reasons, are not as full and complete as I could now wish.

April 29, 1856, I was requested to see Mrs. C., aged 41 years. Found her on a canal boat in the Clark and Skinner Canal, near Perry street bridge. Had abortion three times previous to this. Has had four living children. Her first abortion was produced intentionally; the last occurred in October, 1855, some six months ago. Says she has menstruated regularly since until the second week in April, For ten days past has had light colored discharge. Three days ago it became offensive, and is now almost intolerable. Passes shreds and pieces of membrane. Suffers intense pain. Tenderness of the abdomen, uterus and vagina. Treatment—warm vaginal injections, fomentations to the abdomen and morphia in full doses.

30th, 9 A. M. Found her about the same—a little less tenderness and pain for the last three hours. Enemas of warm water were added to the previous treatment for the purpose of relieving the bowels.

3 P. M, No perceptible change.

9 P. M. Found her in great distress, and still discharging shreds of membrane, clots, etc., partially decomposed, and so offensive that it was almost impossible to remain in the room. Ordered blister to the abdomen, and gave morphia and also chloroform one part to two of sulph. ether, pro. re nata. Besides these she had also taken gum opii in quite large doses, which she had procured on her own responsibility.

May 1st. About the same. Dressed blister with simple cerate sprinkled freely with morphia, and ordered the anodynes in still larger doses.

2d, 9 A. M. But little change. During the last twelve hours she has taken sixty grains of opium, (five grains every hour) two grains morphia, and chloroform in as large doses as was thought consistent with safety. Yet she found no relief from pain nor had she slept for a moment. She now appeared weak. Gave two grains quinine every six hours in addition to former treatment, which was continued until the 7th, when there was but slight if any improvement perceptible.

9th. I find her not as well this morning, suffering great pain.

3 P. M. Dr. J. P. White was called in consultation. After a thorough examination could not make a satisfactory diagnosis. Advised a continuance of previous treatment. Discharge still offensive.

10th. Fonnd her more comfortable this morning, and able to be moved from the boat to a house on Exchange street.

11th, 10 A. M. Patient bore removal well, and is much more comfortable.

4 P. M. Drs. White, Rochester, and Storer of Boston, who, visiting at Dr. White's, kindly volunteered to see her with us in consultation. Being in a great measure relieved from pain she gave the history of her former abortions more definitely. Says the first was produced intentionally twelve years ago. The second, six years later. The third she thinks was about the fourth of October, 1855. Menstruated regularly after that until March following, when she had severe flooding. Five weeks later, in the fore part of April, flowed two days, when the discharge became lighter colored and continued until the 27th, when it became exceedingly offensive and accompanied with severe pain in the abdomen and back. At this time I first saw the patient and commenced treating her as already stated. The case was of more professional interest in many of its details than I may be able at this time to invest it. Dr. White had previously examined thoroughly, and at this time Drs. Rochester and Storer made careful recto vaginal examinations, but failed to determine the character of the tumor, which was pressing the uterus apparently back and upwards. I shall never forget my disappointment at this failure, for Dr. Storer at the solicitation of Dr. White, had very kindly consented to go with us and unravel the mystery. So the diagnosis was not yet satisfactorily made out. That there was a large tumor, occupying nearly the whole pelvis was plain, but its full extent and exact character was yet to be determined. Then again all attempts at exploration with the uterine sound were futile. There was an aperture on the posterior wall of the vagina, which we supposed to be what there was of the os uteri, displaced and changed by the peculiar character and position of the pelvic tumor. The anterior part of the vagina seemed to end in a short culde sac. As I have said, we could find what we supposed to be the os uteri. Yet we could not succeed in making the least exploration and the reasons will appear hereafter. All agreed upon one thing, and that was, an unfavorable prognosis.

May 12. Not so well. Suffers from pain as heretofore. A little wine was now allowed in addition to former treatment.

13th, 14th, 15th. No particular change. The same suffering from pain and the same sustaining and anodyne treatment.

16th. Dr. White saw her with me to-day. The tumor is distinctly tracable in left side of abdomen by external manipulation. Yet most tender and sensitive to the touch internally on the right side of the pelvis. Dr. White pierced the tumor from below with an exploring trochar, and when requested to withdraw it I found it firmly grasped, and had to use strong force to withdraw it. Nothing but a little blood followed its extraction. I should say in this connection that Dr. White had previously stated his opinion that the tumor was fibrous in character, and this experiment clearly confirmed it. The absence of fluid discharge and the firm grasp upon the instrument were the evidences. Gin sling and essence of beef were added to the former treatment.

18th, Bowels constipated and somewhat uncomfortable. Ordered a dose Oil Racini.

19th. Oil operated well. Discharge of blood nearly ceased.

20th, Had nine dejections since yesterday. Complains of weakness, but upon the whole appears much better. Has some discharge of blood.

21st. Found her feeling much better, and sitting at the table taking her tea. The flowing had ceased.

22d. Had eight dejections since yesterday; feels quite comfortable; appetite improving; tumor apparently growing less and pressing upon the perineum; it was hard and less tender to the touch. Ordered iodide potass ʒii, ext. conii ʒi, syrup lemon ℥i, aqua ℥iii, M. A teaspoonful to be taken three times daily, and as little opium as possible. From this time she continued to improve daily.

June 9th. Patient was able to walk from her residence on Exchange street to the Clarendon Hotel, and to market. Continued last prescription. Continued to convalesce, and was soon able to attend to her domestic duties, Saw no more of her until December 30th, when I was called to see her again. Found the tumor in the same position, and apparently larger than when last examined.

Dec. 31st. As Dr. White had taken much interest in the case I again invited him to see her. He confirmed my opinion that it was increasing in size. Could not introduce an instrument in what was supposed to be the os. Prescribed the following: Iodide potass ʒiv, Iodine ʒi, syrup sarsaparilla ℥viii, M. A teaspoonful to be taken three times daily, also vaginal injections and rest. From this time she has been able to be about and do her work most of the time. She has had occasional flowing, and some times it would be very severe for three or four days. Sometimes I would not see her for a year or two. Whether she had other medical attendance during such intervals I am unable to say. On the 27th of September, 1864, she was taken sick and sent for me. Being too unwell to attend, Dr. J. F. Miner was called and attended her until October 2d, when I took charge of the case. I found her in great distress, severe and persistent; vomiting at times stercoraceous matter; bowels tympanitic, very tender and constipated; had no passage for several days; pulse scarcely perceptible at the wrist; feet and hands cold; pain in abdomen intense. Dr. Miner, by note, and before I left, in person, explained or gave a history of the case and treatment during his attendance. We agreed the case was hopeless, and attempted only to palliate the symptoms. Gave morphine and chloroform *pro re nata.*

Oct. 3d. Found her in *articulo mortis.* Died at one P. M. In accordance with her desire expressed on former occasions, and also repeated just before her death, I arranged for a *post mortem,* which was made forty-five hours after death, by Dr. C. C. F. Gay, and his student, Mr. Damback. Drs. White and Rochester were also present. On opening the abdomen the omentum appeared unusually thin and congested. Peritoneal inflammation strongly marked in the cavity and on the intestines, with considerable pus and serum throughout the lower part of abdomen. In some parts strong adhesions. There was an ovarian cyst which at first sight was supposed to be the bladder distended with urine, and which it very much resembled before removal, It contained about thirty ounces of fluid. The walls of the uterus were thick and cartilaginous. On the anterior part of the uterus was a fibro cartilaginous tumor about three inches in diameter, and on the inside another about two inches. The vagina had a stricture barely large enough to admit the point of the finger. This prevented all means of reaching the os tincæ by finger or instrument, and might very naturally be mistaken for the os as it certainly was during life. The uterus with the tumors and cyst unopened, were preserved for presentation to the

Medical Association. It was placed in a secure corner, as I supposed, but mysteriously disappeared during my absence for a day or two from the city. As it was placed in a new, covered tin pail, obtained for the purpose, the thief no doubt supposed it contained something valuable, and so it did, but not for whoever took it away. If they drank the fluid, or partake of the solid contents, may they enjoy the luxury. Its loss deprived the Society of the examination of a very interesting specimen of uterine disease, originating undoubtedly from the effect of inflammatory action. In this case we see an exemplification of the evil effects of criminal abortion. In the absence of positive evidence that any attempts were made for the purpose, yet we cannot account in any other way for the violent symptoms met with in the progress of the case. The violent, intense pain, the offensive discharge, the stricture of the vagina and its adhesions clearly indicate what must have been the inciting cause, commencing perhaps as far back as the first abortion, which she admitted was intentionally produced. From my first visit in 1856, until death closed the scene, this has been a case of great interest to me and those whose counsel and advice I have availed myself. To Dr. White particularly, who for so many years voluntarily gave his time and attention whenever called upon, I feel under lasting obligations. There are several very unusual circumstances in this case, and no one can fully appreciate them from the imperfect manner in which I have presented them to the readers of the Journal. First, there was the excessive, long continued pain and offensive discharge, and when I say it was *unusual*, it expresses but a tithe of the reality. I have had some experience in such cases, when occurring from both accident and design, but nothing that bore any comparison to this.

2d.—Fibrous tumors I presume are not very unusual, but are they often found both internal and external at the same time? In this case it was not only so, but the body of the uterus had also degenerated into the same condition. As to their cause generally, much obscurity exists. In this case it may have arisen from contusion or injury from attempts to produce abortion. And this was undoubtedly the cause of the stricture of the vagina. I once attended a woman confined by a midwife, who complained that she had used greats violence in the first stage of labor, and that she felt as if torn to pieces. On examination I found the vagina badly ruptured, and had to remove some protruding portions as large as my three fingers. She recovered, and Iwas allowed to make an examination. The vagina was obliterated, the walls solidly united. all beyond a sealed book, and the danger of a similar accident removed forever.

Solid tumors seldom if ever ulcerate; yet flooding may occur, and did often in this case, Why so much when the patient enjoyed otherwise good health, I am unable to explain. Nor do I believe that such complications often occur, although we find them on record. The ovarian cyst was not opened. I have no doubt that was also produced from the same cause as the tumors. I regret now it is lost that we did not open it so that I could speak of its contents.

The large amount of opium administered is worthy of notice. As I have stated she had severe pain, to allay which, I found ordinary doses of no avail, so I ordered five grains every hour until she was relieved. When I saw her twelve hours after she had taken sixty grains opium, two grains morphine, equivalent to twelve grains opii, and two ounces chloroform had been inhaled also, without producing the least symptom of narcotism or even sleep, neither had it perceptibly alleviated the pain. In this we have an example of the tolerance of anodynes in severe suffering.

